# Clinical practice guideline for anterior segment dysgenesis

**DOI:** 10.1007/s10384-025-01297-x

**Published:** 2026-04-09

**Authors:** Research on rare and intractable diseases, Health, Labour and Welfare Sciences Research Grants Clinical Practice Guideline Development Committee for Aniridia of the “Research group on establishing standardized diagnosis and treatment of Intractable corneal diseases”

**Affiliations:** Osaka, Japan

## Preface

Anterior segment dysgenesis is a congenital anomaly in which the main abnormal findings are localized to the anterior eye (cornea, iris, angle). It collectively encompasses posterior embryotoxon, Axenfeld anomaly, Rieger’s anomaly, posterior keratoconus, Peters anomaly, sclerocornea, and anterior staphyloma. Many cases are sporadic, but there are also cases with autosomal recessive or autosomal dominant inheritance, and gene mutations such as *PAX6, PITX2, CYP1B1*, and *FOXC1* are reported. Anterior segment dysgenesis may be unilateral or bilateral; according to Japanese statistics, approximately 75% of cases are bilateral. Anterior segment dysgenesis with corneal opacity is often accompanied by form-vision deprivation amblyopia and generally has a poor prognosis for visual acuity, with many documented cases of severe visual impairment.

This disease is listed as a designated intractable disease based on the Act on Medical Care for Patients with Intractable Diseases (Intractable/Rare Diseases Act), and its diagnostic criteria and severity classification were prepared by the research group on Intractable corneal diseases on establishing standardized diagnosis and treatment. We have created these clinical practice guidelines in line with the Medical Information Network Distribution Service (Minds) format to provide high-quality medical care for patients with anterior segment dysgenesis. Minds is a project operated by the Japan Council for Quality Health Care, commissioned by the Ministry of Health, Labour and Welfare. According to Minds, clinical practice guidelines are defined as *“Documents presenting the optimum recommendation to support the decision-making of patients and healthcare professionals, from the systematic reviews of evidence, their overall assessment, and the balance between benefits and harms, etc., for medical practices of high clinical importance.”* This study group aimed to create evidence-based guidelines for important clinical issues in the treatment of anterior segment dysgenesis, rather than authority-based methodologies that have gathered the opinions of experts. Evidence was collected in an established method to form a systematic review (SR) that was evaluated and summarized as a whole; recommendations for important clinical issues were summarized based on this evaluation.

To ensure an unbiased judgment process in the development of clinical practice guidelines, it is recommended that tasks be divided and carried out by three groups: clinical practice guidelines supervisory committee, clinical practice guidelines development group, and systematic review team. The clinical practice guidelines supervisory committee supervises the overall development. The clinical practice guidelines development group is responsible for defining the scope and identifying the key clinical questions (CQs). The systematic review team conducts systematic reviews based on the identified CQs and evaluates the overall body of evidence. The organizational structure and roles in the development of this clinical practice guideline are detailed in Table 1.

**Table 1:** Guideline Development Organizations

(1) Main body for the development of the clinical practice guideline	Name of academic society/study group		Research program on rare and intractable diseases, Health, Labour and Welfare Sciences Research Grants; the “Research group on establishing standardized diagnosis and treatment of intractable corneal diseases”	
	Name of related/collaborating academic society		Japanese Ophthalmological Society	
	Name of related/collaborating academic society		Japan Cornea Society	
	Name of related/collaborating academic society		Japanese Association of Pediatric Ophthalmology	
	Name of related/collaborating academic society		Japan Glaucoma Society	
(2) Clinical practice guideline supervisory committee	Representative	Name	Affiliation/specialty	Role
	〇	Kohji Nishida	Department of Ophthalmology, The University of Osaka Graduate School of Medicine/Ophthalmology	Supervision of guideline development
		Akira Murakami	Department of Ophthalmology, Juntendo University Graduate School of Medicine/Ophthalmology	Instruction on guideline development
		Noriyuki Azuma	Department of Ophthalmology and Laboratory for Visual Science, National Center for Child Health and Development/Ophthalmology	Instruction on guideline development
		Jun Shimazaki	Department of Ophthalmology, Tokyo Dental College, Ichikawa General Hospital/Ophthalmology	Instruction on guideline development
		Kazunori Miyata	Miyata Eye Hospital, Medical Corporation Meiwakai/Ophthalmology	Instruction on guideline development
		Masakazu Yamada	Department of Ophthalmology, Kyorin University School of Medicine/Ophthalmology	Instruction on guideline development
		Chie Sotozono	Department of Ophthalmology, Kyoto Prefectural University of Medicine/Ophthalmology	Instruction on guideline development
		Atsushi Shiraishi	Department of Ophthalmology, Ehime University School of Medicine/Ophthalmology	Instruction on guideline development
		Shigeto Shimmura	Department of Ophthalmology, Keio University School of Medicine/Ophthalmology	Instruction on guideline development
		Tomohiko Usui	Department of Ophthalmology, The University of Tokyo Hospital/Ophthalmology	Instruction on guideline development
		Yoshinori Oie	Department of Ophthalmology, The University of Osaka Graduate School of Medicine/Ophthalmology	Instruction on guideline development
(3) Secretariat for clinical practice guideline development	Representative	Name	Affiliation/specialty	Role
	〇	Yoshinori Oie	Department of Ophthalmology, The University of Osaka Graduate School of Medicine/Ophthalmology	Review of public comments, disclosure of guideline
		Nozomi Nishida	Department of Ophthalmology, The University of Osaka Graduate School of Medicine/Ophthalmology	Review of public comments, disclosure of guideline
(4) Clinical practice guideline development group	Representative	Name	Affiliation/specialty	Role
	〇	Masakazu Yamada	Department of Ophthalmology, Kyorin University School of Medicine/ Ophthalmology	Guideline development
		Kohji Nishida	Department of Ophthalmology, The University of Osaka Graduate School of Medicine/Ophthalmology	Guideline development
		Noriyuki Azuma	Department of Ophthalmology and Laboratory for Visual Science, National Center for Child Health and Development/Ophthalmology	Guideline development
		Kazunori Miyata	Miyata Eye Hospital, Medical Corporation Meiwakai/Ophthalmology	Guideline development
		Chie Sotozono	Department of Ophthalmology, Kyoto Prefectural University of Medicine/Ophthalmology	Guideline development
		Shigeto Shimmura	Department of Ophthalmology, Keio University School of Medicine/Ophthalmology	Guideline development
(5) Systematic review team	Representative	Name	Affiliation/specialty	Role
	〇	Tomomi Yamada	Department of Medical Innovation, The University of Osaka Hospital/Biostatistics	Supervision of systematic review
		Chika Shigeyasu	Department of Ophthalmology, Kyorin University School of Medicine/Ophthalmology	Systematic review
		Ryohei Nejima	Miyata Eye Hospital, Medical Corporation Meiwakai/Ophthalmology	Systematic review
		Yosai Mori	Miyata Eye Hospital, Medical Corporation Meiwakai/Ophthalmology	Systematic review
		Yuichi Uchino	Department of Ophthalmology, Keio University School of Medicine/Ophthalmology	Systematic review
		Hiroto Mitamura	Department of Ophthalmology, Keio University School of Medicine/Ophthalmology	Systematic review
		Miki Omoto	Department of Ophthalmology, Keio University School of Medicine/Ophthalmology	Systematic review
		Yoko Ikeda	Department of Ophthalmology, Kyoto Prefectural University of Medicine/Ophthalmology	Systematic review
		Hiroyuki Kurakami	Department of Medical Innovation, The University of Osaka Hospital/Statistics	Systematic review
(6) External review committee	Representative	Name	Affiliation/specialty	Role
		Yuichi Hori	Department of Ophthalmology, Toho University Omori Medical Center/Ophthalmology	Review of guideline
		Toshiyuki Ojima	Department of Community Health and Preventive Medicine, Hamamatsu University School of Medicine/Public health and epidemiology	Review of guideline

In these clinical practice guidelines, we have summarized the evidence and made recommendations for three clinical questions (CQs) considered important in clinical practice (Table2). Randomized controlled trials (RCTs) and other studies with high evidence levels have not been conducted on rare diseases such as anterior segment dysgenesis, and strong recommendations could not be made for any of the CQs. For this reason, there are still some controversial aspects in terms of consistency and clarity. Therefore, we appreciate the readers’ understanding that the purpose of the clinical practice guidelines is not to provide textbooks and rules but to help medical practice. We hope these guidelines will help patients and healthcare professionals to select the best practice.

**Table 2:** Guideline Summary

CQ no.	CQ	Summary and recommendation	Level of recommendation
1	What tests are useful for diagnosing the disease type of anterior segment dysgenesis?	Ultrasound biomicroscopy (UBM) and anterior segment optical coherence tomography (OCT) are suggested to diagnose the disease type in patients where anterior segment dysgenesis is suspected based on clinical findings.	Weak: suggest “to implement”
2	Is surgical intervention more effective than the natural course for corneal opacity in anterior segment dysgenesis?	There are no reports comparing surgical treatment for corneal opacity in anterior segment dysgenesis during the natural course. Surgical treatment may provide clear corneal healing in the short term, but the long-term prognosis is unknown. Owing to the risk of complications associated with intraoperative vitrectomy and lensectomy, as well as development of postoperative secondary glaucoma, surgery is not recommended.	Weak: suggest “not to implement”
3	What tests are useful for early detection and management of secondary ocular complications of anterior segment dysgenesis?	It is necessary to understand that the criteria for suspecting glaucoma in children are different from those in adults. As useful tests for early detection and management of secondary glaucoma in anterior segment dysgenesis, we suggest the measurement of corneal diameter and intraocular pressure in non-crying infants, and an intraocular pressure test and visual field test from school age to adulthood. Evaluation of optic disc cupping is important in patients whose fundus can be visualized distinctly.	Weak: suggest “to implement”

Research on rare and intractable diseases, Health, Labour and Welfare Sciences Research Grants

The research group of “Intractable corneal diseases on establishing standardized diagnosis and treatment”

Masakazu Yamada, Co-Investigator

Noriyuki Azuma, Co-Investigator

Kohji Nishida, Principal Investigator

## List of Authors

**Table:** 

Chairperson Kohji Nishida Department of Ophthalmology, The University of Osaka Graduate School of Medicine Committee members (In alphabetical order) Noriyuki Azuma Department of Ophthalmology and Laboratory for Visual Science, National Center for Child Health and Development Yoko Ikeda Department of Ophthalmology, Kyoto Prefectural University of Medicine Hiroyuki Kurakami Department of Medical Innovation, The University of Osaka Hospital (Statistics) Hiroto Mitamura Department of Ophthalmology, Keio University School of Medicine Kazunori Miyata Miyata Eye Hospital, Medical Corporation Meiwakai Yosai Mori Miyata Eye Hospital, Medical Corporation Meiwakai Ryohei Nejima Miyata Eye Hospital, Medical Corporation Meiwakai Yoshinori Oie Department of Ophthalmology, The University of Osaka Graduate School of Medicine Miki Omoto Department of Ophthalmology, Keio University School of Medicine Chika Shigeyasu Department of Ophthalmology, Kyorin University School of Medicine Shigeto Shimmura Department of Ophthalmology, Keio University School of Medicine Chie Sotozono Department of Ophthalmology, Kyoto Prefectural University of Medicine		Yuichi Uchino Department of Ophthalmology, Keio University School of Medicine Masakazu Yamada Department of Ophthalmology, Kyorin University School of Medicine Tomomi Yamada Department of Medical Innovation, The University of Osaka Hospital/Biostatistics External review committee members Yuichi Hori Department of Ophthalmology, Toho University Omori Medical Center Toshiyuki OjimaDepartment of Community Health and Preventive Medicine, Hamamatsu University School of Medicine/Public Health and Epidemiology Approved by Japanese Ophthalmological Society Japan Cornea Society Japanese Association of Pediatric Ophthalmology Japan Glaucoma Society Collaborators Noriaki Akai Life Sciences Library, The University of Osaka Nozomi Nishida Department of Ophthalmology, The University of Osaka Graduate School of Medicine

Medical Diagram
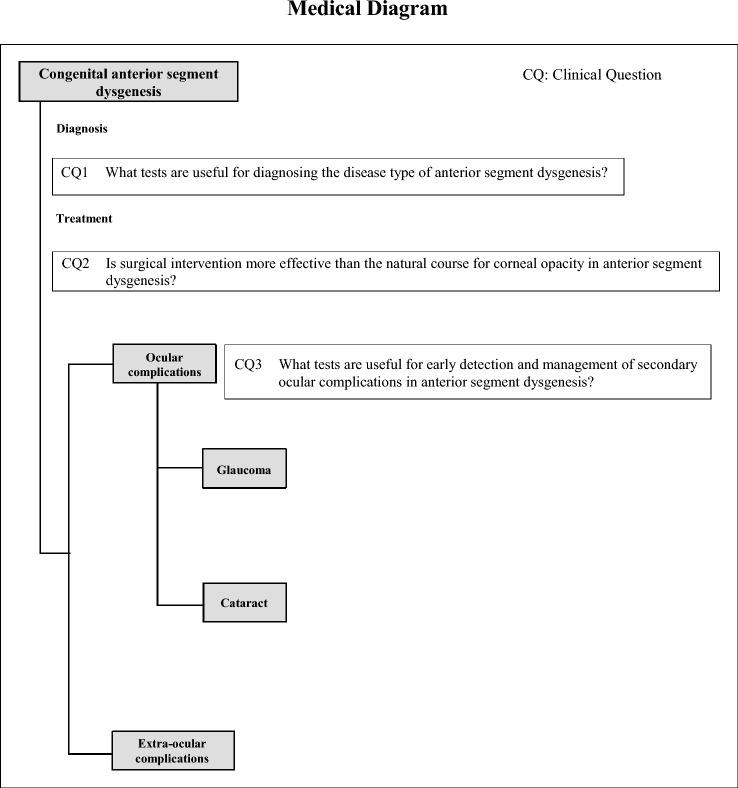


## Key to important terminology

**Table:** 

Term	Description
Axenfeld anomaly	A type of anterior segment dysgenesis characterized by thickening and anterior displacement of the Schwalbe line in the angle and anterior adhesion of the iris. It is often associated with glaucoma.
Sclerocornea	A congenital anomaly that resembles the sclera, with opacification of some or all of the cornea. This is also called a cornea plana.
Form-vision deprivation amblyopia	Amblyopia results from the inability to obtain a clear retinal image because of obstruction in visual pathways, including corneal opacity, cataract, ptosis, etc.
Posterior keratoconus	A congenital anomaly with a local defect in the posterior corneal stroma. The lesion is indented locally, and there is increased curvature of the posterior corneal surface.
Posterior embryotoxon	A congenital anomaly in which the Schwalbe line protrudes toward the anterior chamber, thickens, and deviates forward. It presents as ring-shaped cloudiness around the cornea.
Photophobia	Light sensitivity
Anterior segment dysgenesis	Abnormal eye developments, with the main abnormal findings localized to the anterior segment of the eye (cornea, iris, angle).
Anterior staphyloma	A congenital anomaly in which the cornea protrudes forward with the iris tissue. The thinned cornea and iris are adherent.
Peters anomaly	A type of anterior segment dysgenesis that causes corneal opacity consistent with defects in the corneal endothelium and Descemet’s membrane. It may be accompanied by abnormal anterior chamber angle or cataract. Attention should be paid to glaucoma complications.
Rieger’s anomaly	A type of anterior segment dysgenesis with protrusion of the Schwalbe line in the angle into the anterior chamber, iris adhesion, and iris atrophy. This often causes glaucoma.
Low vision care	Habilitation/rehabilitation for visually impaired persons and children.

## Details of recommendations and explanations

### Throughout the guidelines

These clinical guidelines were created in accordance with the “Minds Manual for Guidelines’ Development 2017.” Important clinical issues were determined by reviews conducted by the guidelines’ development committee. Those that could be presented as recommendations were taken up in the form of clinical questions (CQs, and a systematic review (SR) was conducted for each CQ outcomes. A recommendation was prepared based on the results.

**Table:** 

**Clinical question**	
Each CQ is based on important clinical issues addressed in the clinical practice guidelines and developed by extracting the components (patient/population, intervention, comparison and outcomes) of relevant questions that should be answered within the guidelines. A CQ presents the extracted components in a question style. In these clinical practice guidelines, important clinical issues were divided into three items—diagnosis, treatment, and ocular complications—to form three CQs.	

**Table:** 

**Presentation of recommendations**	
Based on the SR results of each CQ, recommendations were decided through the deliberation of the guidelines development group by taking into account the strength of evidence, balance between benefits and harms, diversity of patient values and intentions, and economic perspectives related to outcomes. We have summarized the limited evidence and present the best policy as recommendations even for issues for which recommendations based on scientific evidence are difficult owing to the nature of the rare diseases.	

**Table:** 

**Strength of recommendation**	
The strength of the recommendations was determined by the guidelines’ development group according to the method defined in the scope and is presented in the following four categories according to the direction and strength of the recommendation: Strongly recommended to implement Weakly recommended suggested to implement Weakly recommended suggested not to implement Strongly recommended not to implement	

**Table:** 

**Strength of evidence for the Clinical question**	
A summary of the evidence for each CQ was presented by integrating the “strength of evidence (total evidence)” evaluated for each outcome. The definitions of each level of strength of evidence, A to D, are as follows. In principle, the strength of evidence was judged to be D (very weak) or C (weak) when there were only case reports or case series. A (strong):Strong confidence in the estimated effect B (moderate):Moderate confidence in the estimated effect C (weak):Limited confidence in the estimated effect D (very weak):The estimated effect is almost uncertain	

**Table:** 

**Development process of the recommendation**	
The background to the presentation of recommendations based on the CQs is provided.	

**Table:** 

**Summary of SR report**	
A commentary on the determination of the overall strength of the evidence is provided along with the results of the qualitative SR.	

**Table:** 

**References**	
The list of references used for the SR is provided.	

## Chapter 1

### Scope

The clinical practice guidelines were prepared by revising the diagnostic criteria and severity classification of anterior segment dysgenesis. Anterior segment dysgenesis is a major cause of congenital corneal opacity, but its clinical picture is diverse and its severity varies greatly from case to case. The diagnostic criteria and severity classifications are based on those proposed by the study group of the Ministry of Health, Labour and Welfare, and answers are provided based on evidence-based medicine for questions that require clinical judgment (i.e., CQs). However, since the disease is rare, there are few clinical studies with a high level of evidence both in Japan and overseas. For this reason, there are many parts of this clinical practice guidelines that have had to rely on the views of this committee, such as the national survey conducted by the sub-investigators and the database of anterior segment dysgenesis.

These clinical practice guidelines are intended to be the standard guide for clinical practice in Japan at present; however, in actual medical care, the treatment strategy should only be determined after fully considering the individual patient’s circumstances and pathological conditions. These clinical practice guidelines do not enforce the selection of treatment or restrict physicians' decisions. These guidelines will be revised as appropriate in the future as diagnosis and treatment methods improve.

#### I. Clinical characteristics

**Figure 1:**
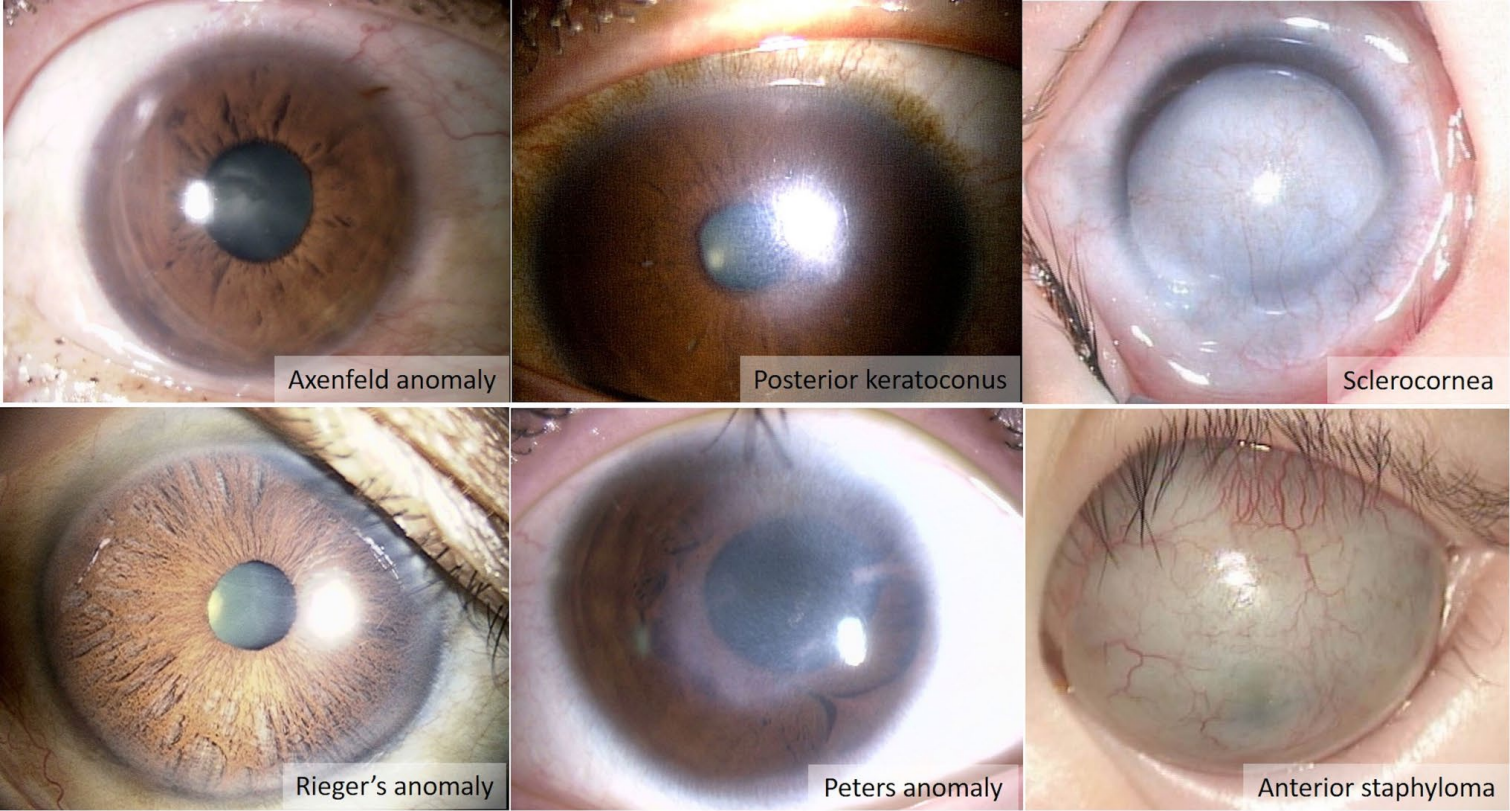
Clinical types of anterior segment dysgenesis

Anterior segment dysgenesis, in which the main abnormalities among congenital ocular anomalies are confined to the anterior segment (cornea, iris, angle), collectively includes posterior embryotoxon, Axenfeld anomaly, Rieger’s anomaly, posterior keratoconus, Peters anomaly, sclerocornea, and anterior staphyloma (Figure 1).

#### II. Epidemiological characteristics

The incidence of anterior segment dysgenesis accompanied by corneal opacity in Japan is estimated to be 1 in 12,000–15,000 births, and the annual number of patients is approximately 70–90 according to a study for understanding the current status of congenital corneal opacity and establishing a diagnostic method (2009 MHLW Sciences and Research Grant for the Research on Measures for Intractable Diseases); thus, it can be considered a rare disease [1–3]. However, mild cases without or unclear corneal opacity were not included in this study, and the actual numbers of patients may be higher. In Spain, it is estimated to be 1 in 5,000–6,000 births [4]. There is no difference in occurrence between males and females.

There are many sporadic cases; however, some patients have autosomal recessive or autosomal dominant inheritance [5].

#### III. Pathophysiology

In terms of the development of the anterior segment, the separation of the lens vesicles from the surface ectoderm begins around 5 weeks of embryonic development, followed by the formation of the corneal epithelium, formation of the corneal endothelium at the 6th week of embryonic development (1st wave), formation of the corneal stroma at the 7th week of embryonic development (2nd wave), and formation of the iris stroma at the 8th week of embryonic development (3rd wave), showing continuous development within a short period of time. Each tissue’s origin also varies from the neuroectoderm, surface ectoderm, and neural crest cells, and the developmental abnormalities that occur during this period exhibit various clinical types.

Corneal opacity associated with anterior segment dysgenesis is based on a defect in the cornea’s posterior surface and is derived from abnormal migration of neural crest cells at the 6th week of embryonic development (1st wave) [6–8]. The clinical picture is diverse as secondary abnormalities of the 2nd and 3rd waves may occur, and the above disease groups can be regarded as a spectrum of conditions [9–14]. Gene mutations such as *PAX6, PITX2, CYP1B1*, and *FOXC1* are reported [13, 15, 16].

Anterior segment dysgenesis may be unilateral or bilateral; according to Japanese statistics, approximately 75% of cases are bilateral. When one eye has a Peters anomaly, the other eye also suffers from Peters anomaly in half of the cases, and 20–30% of patients demonstrate other anterior segment dysgenesis disorders such as sclerocornea and anterior staphyloma [2, 5].

#### IV. Clinical symptoms and test findings

1. ** Eye findings**

**Figure 2:**
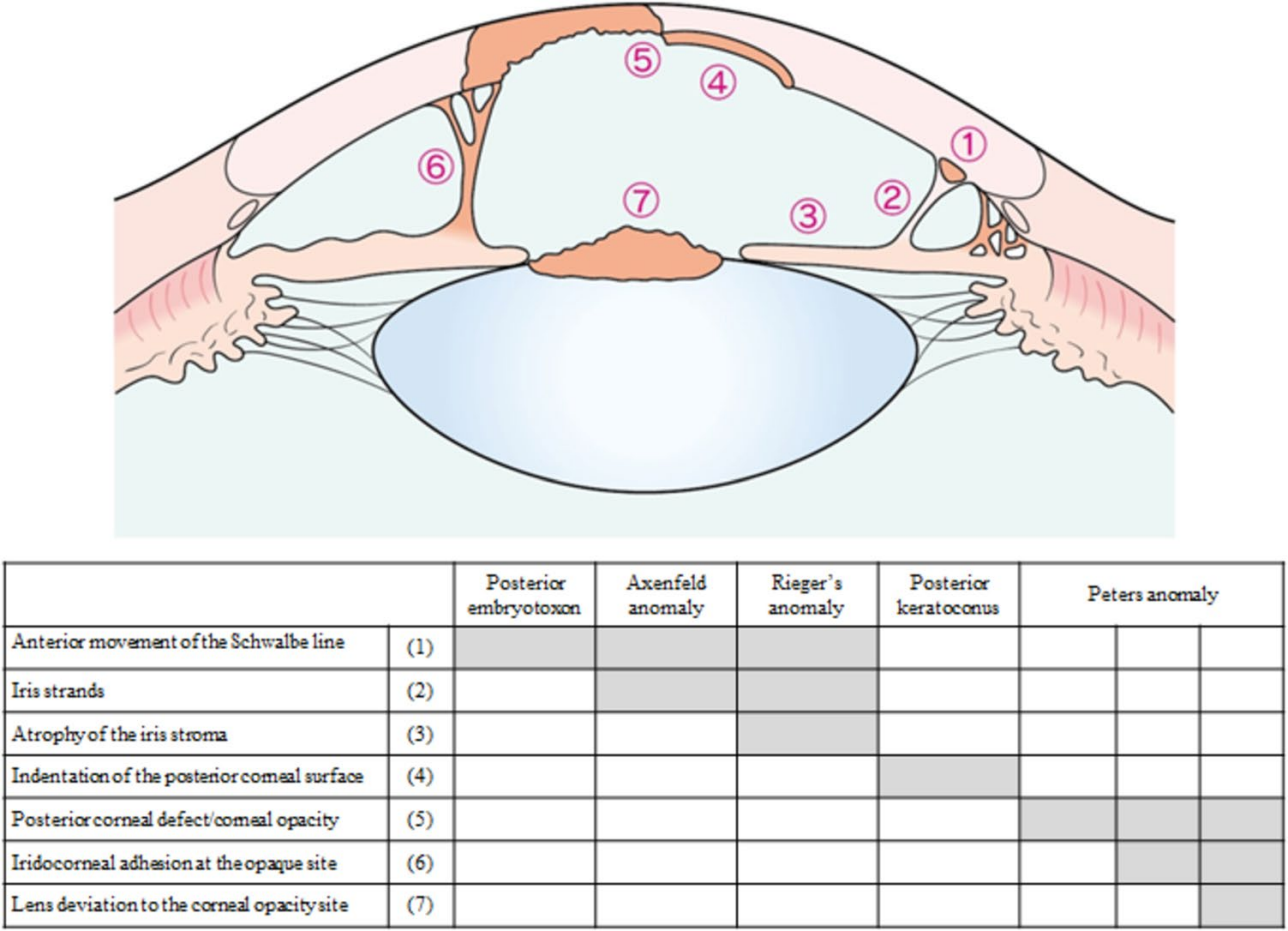
Clinical features and disease types of anterior segment dysgenesis. Peters anomaly results in (5) posterior corneal defect in the center and (6) iris strands (Peters anomaly type I). When accompanied by (7) anterior movement of the lens or cataract, it is classified as Peters anomaly type II.

Key findings of anterior segment dysgenesis include (1) anterior movement of the Schwalbe line, (2) iris strands, (3) atrophy of the iris stroma, (4) indentation of the posterior corneal surface, (5) posterior corneal defect/corneal opacity, (6) iridocorneal adhesion at the opaque site, and (7) anterior movement of the lens to the corneal opacity site (Figure 2) [9, 10, 17].

2. **Systemic findings**

Bilateral cases are more likely to have systemic complications than unilateral cases [18], and 20–30% of patients have various systemic abnormalities such as cardiovascular abnormalities, neurological disorders, developmental disorders, and multiple systemic malformations [2, 5, 19]. Embryologically, many midline tissue abnormalities have a common origin of neural crests [6, 20, 21].

Of these, complications with dental abnormalities, facial bone abnormalities, umbilical abnormalities, pituitary lesions, etc., are known as Axenfeld-Rieger syndrome. *PITX2* gene abnormalities have also been reported, indicating autosomal dominant inheritance. Those with cleft lip/palate, growth disorder, developmental disorder, or congenital heart disease are classified as having Peters plus syndrome. A mutation in the *B3GALTL* gene has been reported in this syndrome, indicating autosomal recessive inheritance.

#### V. Diagnosis and tests

Anterior segment dysgenesis should be considered if corneal opacity of all or part of one or both eyes is present in neonates or infants and is accompanied by strands that continue from the posterior corneal surface to the iris or posterior corneal defects.

**Figure 3:**
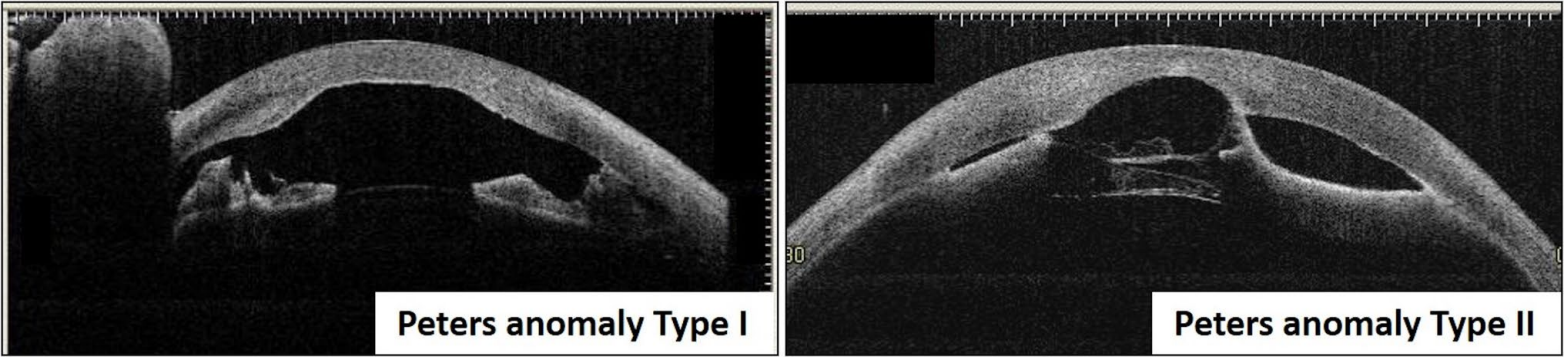
Findings using anterior segment OCT in Peters anomaly

The basic finding in Peters anomaly, which is the most common, is central corneal opacity that coincides with posterior corneal defects; however, it may also present with extensive diffuse opacity. Whenever slit-lamp examination can confirm iris strands, anterior movement of the lens, or cataract, it will lead to a definitive diagnosis. Peters anomaly and sclerocornea are associated with severe corneal opacity. When it is difficult to observe by slit-lamp examination, anterior segment OCT or anterior segment UBM is useful (Figure 3) [22–26].

In sclerocornea, most or all of the cornea is formed of white tissue that is difficult to distinguish from the sclera, and the surface is covered with epithelial tissue with blood vessels. Normal corneal tissue may be observed in the peripheral or central parts. In sclerocornea, similar to Peters anomaly, anterior segment OCT demonstrates continuous strands in the iris from the posterior corneal surface, posterior corneal defects, or lens abnormalities. Anterior staphyloma has extensive thinning of the corneal stroma and loss of the anterior chamber, and the anterior movement of the iris causes it to be pushed up behind the corneal epithelium by intraocular pressure [23].

B-mode ultrasonography and, if possible, fundus examination should be conducted to detect abnormalities in the posterior segment of the eye. Periodic intraocular pressure measurements are recommended as secondary increases in intraocular pressure can occur. If ophthalmologic examinations, including intraocular pressure examinations, are difficult to perform in young children, examinations should be planned under general anesthesia.

Evaluation of visual function by refraction /visual acuity tests is desirable, but evaluation of refraction often depends on subjective tests owing to corneal opacity. In cases where visual acuity tests are not possible, such as in young children and in those with developmental disorders, it may be estimated from ophthalmologic examination findings, visual reactions, and daily behavior.

#### VI. Treatment and prognosis

In Peters anomaly, corneal opacity itself often improves with growth [27, 28], but does not change in the sclerocornea and anterior staphyloma. Since both cases are often accompanied by form-vision deprivation amblyopia, it is necessary to start amblyopia treatment from an early stage, especially with unilateral cases. Nonetheless, visual acuity prognosis is generally poor. Vision in Peters anomaly is less than 0.1 and less than 0.01 in 60% and ≥40% of cases, respectively, indicating that many patients exhibit severe visual impairment. In sclerocornea and anterior staphyloma, almost all patients have visual acuity of less than 0.01[2].

Penetrating keratoplasty (PKP) is sometimes performed for Peters anomaly and sclerocornea, but postoperative visual acuity often depends on disease severity [18, 19, 29]. When performing keratoplasty in infants, surgical techniques and postoperative management are difficult [19, 30–35]. Complications such as corneal graft rejection, cataract, inoperable retinal detachment, and phthisis bulbi are more common in infants than in adults [30, 36]. Consequently, surgery is rarely conducted in Japan [27, 37]. For anterior staphyloma, enucleation or evisceration may be performed because of exophthalmos or corneal perforation.

In general, anterior segment dysgenesis is prone to secondary glaucoma at school age to adolescence [9, 27, 38], and attention should be paid to intraocular pressure management to maintain visual function. There are also cases where secondary lens opacification (cataract) occurs [9, 39].

Since the degree of corneal opacity varies from case to case, the degree of visual dysfunction also varies; however, it is important to provide low vision care to develop and utilize the remaining visual function. Corneal opacity and irregular astigmatism tend to reduce contrast sensitivity and induce photophobia in anterior segment dysgenesis; therefore, it is necessary to correct refraction if possible and pay special attention to room lighting [40].

#### VII. Flow of medical care and treatment

When considering anterior segment dysgenesis as an intractable disease, the presence or absence of visual impairment that affects daily life and school attendance, its degree, and the need for regular medical care become important.

From this point of view, we have delineated in the diagnostic criteria that among the anterior segment dysgenesis disorders, the main targets are Peters anomaly, sclerocornea, and anterior staphyloma, which cause severe visual impairment owing to corneal opacity. Visual acuity of the better eye, which has the greatest influence on daily living functions, is used for classification in severity grading. Posterior embryotoxon, Axenfeld anomaly, Rieger’s anomaly, and posterior keratoconus, which are mild cases of anterior segment dysgenesis, are excluded unless they are complicated by glaucoma. To ensure appropriate consideration of patients whose visual acuity worsens because of glaucoma after school age and those requiring frequent visits, we added the following remark: “In grades I to III, if secondary glaucoma is accompanied by a narrowing of the visual field of the better eye, the severity classification is moved up by one level.”

I. **Diagnostic criteria**

Among the anterior segment dysgenesis disorders, these diagnostic criteria mainly target Peters anomaly, sclerocornea, and anterior staphyloma, which cause severe visual impairment owing to corneal opacity. Tests that are particularly useful for diagnosis are slit-lamp examination, anterior segment UBM, and anterior segment OCT.

Diagnostic criteria items:

Applies to the “definite” diagnosis category.

A. Symptoms
  1. Corneal opacity that has been present since birth or infancy
  2. Visual impairment
  3. Photophobia
B. Test findings
  The following observations should be made using slit-lamp examination, anterior segment UBM, or anterior segment OCT, etc.
  1. Bilateral or unilateral, full or partial corneal opacity since birth or infancy
  2. Strands that continue from the posterior corneal surface to the iris or posterior corneal defects
C. Differential diagnosis
  1. In utero infection
  2. Trauma during labor (mainly forceps delivery)
  3. Conditions associated with postnatal trauma, infectious diseases, etc.
  4. Conditions associated with systemic inborn metabolic errors
  5. Congenital corneal dystrophy
  6. Congenital glaucoma
  7. Aniridia
  8. Limbal dermoid
D. Extra-ocular complications
  Dental abnormalities, facial bone abnormalities, congenital hearing loss, intellectual developmental disorder, multiple malformations, etc. (Note 1)
E. Genetic tests
  In most cases, there is no family history. However, there are cases of autosomal recessive inheritance and autosomal dominant inheritance. (Note 2)

<Diagnosis category>

Definite:

1. One or more of the items under A and both B-1 and B-2 are observed.
2. One or more of the items under A is observed, B-1 is observed, and all differential diagnoses in C can be excluded.

Probable:

One or more of the items under A and B-1 are observed, but the differential diagnoses in C cannot be excluded.

1. 20–30% of patients have associated extra-ocular complications.
  Axenfeld-Rieger syndrome: complicated with dental abnormalities, facial bone abnormalities, umbilical abnormalities, pituitary lesions, etc.
  Peters plus syndrome: complicated with cleft lip/palate, growth disorder, developmental disorder, congenital heart disease, etc.
2. *PAX6*, *PITX2*, *CYP1B1*, and *FOXC1* gene mutations have been reported in some cases.

II. Severity grading

Applies to those who meet the criteria for (1) or (2).

(1) Applies to those who are classified as grade III or higher, as shown below.

Grade I: Affected eye is unilateral, the other eye is healthy.
Grade II: Both eyes are affected, and the corrected visual acuity of the better eye is ≥0.3.
Grade III: Both eyes are affected, and the corrected visual acuity of the better eye is between 0.1 and 0.3.
Grade IV: Both eyes are affected, and the corrected visual acuity of the better eye is <0.1.

1. In the Healthy condition the corrected visual acuity is ≥1.0, and no visual field and organic abnormalities are observed in the eye.
2. In grades I to III, if secondary glaucoma is accompanied by narrowing of the visual field of the better eye, the severity classification is moved up by one level.
3. Narrowed vision indicates that the residual visual field at the center is within 20 degrees, with the Goldmann I/4 optotype.
4. If visual acuity cannot be measured in patients such as infants, it should be determined comprehensively based on ophthalmological findings, etc. If visual acuity is judged to be between 0.1 and 0.3, denote the grade as between 0.1 and 0.3. If visual acuity is judged to be <0.1, denote the grade as <0.1.

(2) (2) Applies to those who are rated 3 or higher on the modified Rankin Scale (mRS)(Table3), diet/nutrition scale, or respiratory evaluation scale.

**Table 3:** Japanese version of the modified Rankin Scale (mRS) criteria

Modified Rankin Scale		Points of reference
0	No symptoms	A condition without any subjective symptoms or objective signs
1	Symptomatic but no obvious impairment: Can perform daily work and activities	Despite having subjective symptoms or objective signs, there are no restrictions on work or activities that were performed before symptom onset
2	Mild disability: Not all pre-symptomatic activities can be performed, but the patient can perform daily tasks for personal care without assistance	There are restrictions on work and activities performed before symptom onset, but daily activities can be performed on the patient’s own.
3	Moderate disability: Some assistance is needed but the patient can walk without assistance	Assistance is required for shopping and going out using public transportation, but walking, eating, grooming, going to the toilet, etc., do not require assistance.
4	Moderate to severe disability: Assistance is required for walking and physical demands	Assistance is required for walking, eating, grooming, going to the toilet, etc., but continuous care is not required.
5	Severe disability: Bedridden, incontinent, requires constant care and supervision	Continuous care is required.
6	Death	

Japan Stroke Society version

**Diet/nutrition scale (N)**

0 : No symptoms

1 : Some symptoms such as choking and awkward eating behavior, do not interfere with social and daily life

2 : It is necessary to change the form/texture of food and prepare instruments to assist in eating

3 : Some form of assistance is required for diet and nutrition

4 : Requires supplemental parenteral nutrition (tube feeding, total parenteral nutrition, etc.)

5 ; Relies entirely on parenteral nutrition

**Respiratory evaluation scale (R)**

0 : No symptoms

1 : Although decreased vital capacity is observed, it does not interfere with social and daily life

2 : Symptoms such as mild shortness of breath exist because of respiratory distress

3 : Breathing symptoms interfere with sleep or shortness of breath occurs during activities of daily life such as getting dressed

4 : Requires sputum aspiration or intermittent ventilation aids

5 : Requires tracheostomy or continuous use of ventilation aids

#### VIII. Information described in these clinical practice guidelines

1.Title

Clinical practice guidelines for anterior segment dysgenesis

2. **Purpose**

These guidelines aim to improve the following outcomes:

- Diagnosis of anterior segment dysgenesis
- Treatment of corneal opacity
- Management of glaucoma, a major ocular complication
- Prognosis of visual acuity

3. **Topic**

Diagnosis of anterior segment dysgenesis and clinical management of ocular complications

4. **Expected users, facilities, and medical sites where the guideline may be applicable**

Doctors at ophthalmology departments of university hospitals and regional core hospitals, practitioners at eye clinics, and patients

5. **Relationship with existing guidelines**

There are no existing clinical practice guidelines in Japan.

6. **Important clinical issues**

(1) **Diagnosis of anterior segment dysgenesis**

The degree of corneal opacity varies in anterior segment dysgenesis. The frequency of cataract and glaucoma, the ocular complications of anterior segment dysgenesis, can vary as well. It is necessary to re-evaluate with a large sample size to determine which findings are most helpful in diagnosing anterior segment dysgenesis and whether the current diagnostic criteria are valid.

(2) **Treatment options for corneal opacity**

The basic treatment for corneal opacity, the main symptom of anterior segment dysgenesis, is amblyopia treatment and visual rehabilitation in children from infancy to school age. Keratoplasty may be performed for severe bilateral opacity but is rarely performed in Japan owing to its poor prognosis. The optimal treatment for different degrees of unilateral/bilateral corneal opacity still needs to be determined.

(3) **Management of ocular complications such as glaucoma**

In general, anterior segment dysgenesis may engender secondary glaucoma in patients from school age to puberty, and attention should be paid to intraocular pressure management to maintain visual function. As anterior segment dysgenesis encompasses a wide range of pathological conditions, it is necessary to clarify the frequency and prognosis of glaucoma.

7. **Scope covered by the guideline**

Patients diagnosed with anterior segment dysgenesis

8. **Clinical questions list**

CQ1: What tests are useful for diagnosing the disease type of anterior segment dysgenesis?

CQ2: Is surgical intervention more effective than the natural course for corneal opacity in anterior segment dysgenesis?

CQ3: What tests are useful for early detection and management of secondary ocular complications of anterior segment dysgenesis?

#### IX. Information regarding SR

1. **Search schedule**

Literature search:November–December 2018

Literature screening:December 2018–June 2019

Evaluation of overall evidence and summary:

July–October 2019

2. **Search for evidence**

(1) **Evidence types**

The search included existing clinical practice guidelines, SR/meta-analysis (MA) articles, and individual research articles in this order of priority. RCTs, non-randomized controlled trials, observational studies, and case series were included as individual research articles.

(2) **Database**

The search was conducted using Medline (OvidSP), The Cochrane Library, and Ichushi-Web. In addition, articles that are not stored in these databases were included if cited.

(3) **Basic search strategy**

To fully cover existing clinical practice guidelines and SR/MA articles, etc. A general search was conducted initially to prevent omissions, and an individual search was conducted for each CQ. For all databases, the entire recording period of the database was searched unless otherwise specified.

3. **Inclusion and exclusion criteria for literature**

Existing clinical practice guidelines and SR articles that met the inclusion criteria were given priority. If there were no existing clinical practice guidelines or SR articles that met the criteria, an SR was conducted independently for individual research articles (*de novo* SR). In *de novo* SR, priority was given to RCTs that met the inclusion criteria. If no RCT met the criteria, observational studies were included. Depending on the CQ, case series and case reports were also included.

4. **Evaluation method and summary of evidence**

The assessment of the overall strength of evidence followed the method described in the Minds Handbook for Clinical Practice Guideline Development 2017. The integration of overall evidence was based on qualitative and, where appropriate, quantitative integration.

#### X. From preparation of the recommendations through finalization and release

1. **Basic policy for the preparation of recommendations**

Recommendation decisions are based on the deliberations of the guideline development group. Whenever there was no consensus, a vote was taken. In addition to the strength of evidence and balance between benefits and disadvantages, the diversity of patient values and wishes and economic perspectives were also taken into consideration.

2. **Finalization**

An external review was conducted. Public comments were solicited, and the results are reflected in the final version.

3. **Specific method of external evaluation**

The external review committee members submitted comments individually. The clinical practice guidelines’ development group discussed whether the clinical practice guidelines needed to be modified for each comment and decided on the action to be taken. Similarly, for public comments, the clinical practice guidelines development group discussed the need to modify the clinical practice guidelines for each comment and what actions needed to be taken.

4. **Plan for release**

After the external review was complete and public comments were processed, the clinical practice guideline supervisory committee decided on the final release. The release method was decided after discussions between the clinical practice guidelines development group and clinical practice guidelines supervisory committee.

(Masakazu Yamada, Chika Shigeyasu)

## Chapter 2

### Recommendations

-Diagnosis-

**Table:** 

**Clinical question 1** **What tests are useful for diagnosing the disease type of anterior segment dysgenesis?**	

(Kazunori Miyata, Ryohei Nejima, Yosai Mori)

**Table:** 

**Presentation of recommendations**	

UBM and anterior segment OCT are suggested methods to diagnose disease types in patients where anterior segment dysgenesis is suspected based on clinical findings. Both tests are considered effective in assessing the back surface of the cornea, the angle, and the iris, which are difficult to observe with slit-lamp examination. and it is proposed that they should be performed as tests for diagnosing the disease type. However, in some cases, local or general anesthesia is needed for these examinations.

**Table:** 

**Strength of recommendation**	

Weak: suggest to implement

**Table:** 

**Strength of evidence for the Clinical question**	

C (Weak)

**Table:** 

**Development process of the recommendation**	

For the preparation of recommendations for this CQ, we emphasized the ability to visualize the details of the anterior segment, which are difficult to observe with slit-lamp examination.

Corneal opacity often accompanies anterior segment dysgenesis, and it is considered difficult to diagnose disease types using slit-lamp examination alone. UBM and anterior segment OCT are recommended for analysis of structural abnormalities of the cornea, angle, and iris in patients with corneal opacity. We conducted a literature search on the effectiveness of these examinations.

Previous literature on the effectiveness of UBM and anterior segment OCT in the diagnosis of anterior segment dysgenesis included seven case reports [41–47], six case series [22–24, 48–50], and one cohort study [51]. Among them, eight reported the use of UBM [22, 24, 44–47, 50, 51] and 6 reported the use of anterior segment OCT [23, 41–43, 48, 49].

An SR was performed to evaluate their effectiveness in diagnosing disease type and adverse events.

As anterior segment dysgenesis is a rare condition, there is no high-quality literature, such as preceding clinical practice guidelines, SRs, or RCTs. For this reason, an SR was conducted including case reports, case series, and cohort studies. It must be noted that patient age, nationality, and testing equipment varied in the studies.

In eight reports that mentioned diagnosing disease type using UBM, detailed observation of the anterior chamber was possible using UBM. However, it should be noted that UBM, which is a contact-type test, requires local or general anesthesia.

In six reports that mentioned using anterior segment OCT, a detailed observation of the anterior segment was possible, as with UBM, which could not be observed with slit-lamp examination.

No adverse events were reported for either UBM or anterior segment OCT in any literature considered.

**Table:** 

**Summary of SR report**	

1. **Diagnosis of disease type**

Eight reports that mentioned using UBM for the diagnosis of disease type were included. In all reports, UBM enabled detailed observation of the anterior chamber. Although local or general anesthesia is considered necessary for UBM as it is a contact-type test, no case reports provided detailed explanations on how anesthesia was administered. Dietlein et al. report the effectiveness of UBM examinations under general anesthesia in their case series of patients with congenital glaucoma, including Peters anomaly and Rieger’s anomaly [50]. In a case series reported by Yoshikawa et al., examinations were performed under oral sedation [22]. In addition, Mannino et al. report that patients who cooperated could be examined under local anesthesia using 0.4% oxybuprocaine hydrochloride [51].

Six reports that mentioned using anterior segment OCT, which is a non-contact type test, were included. As with UBM, it was possible to observe the anterior segment in detail using anterior segment OCT, which was not possible with slit-lamp examination. Hong et al. report that examination under general anesthesia during keratoplasty was useful in selecting the surgical procedure [49]. Wang et al. also report that anterior segment OCT was useful for adult men who experienced excessive pain with UBM [41].

The evidence level was judged to be C as there are risks of bias due to variations in patient age, nationality, and equipment used, whereas the reports are at the observational study level.

2. **Adverse events**

Adverse events in the use of UBM and anterior segment OCT when diagnosing anterior segment dysgenesis have not been reported in any literature reviewed. The risk of local anesthesia is low, and that of general anesthesia is also considered low when conducted under appropriate control.

Based on the above, we conclude that UBM and anterior segment OCT are useful in the diagnosis of disease type in patients suspected of anterior segment dysgenesis based on clinical findings and that the risk of adverse events is low.

–Treatment-

**Table:** 

**Clinical question 2** **Is surgical intervention more effective than the natural course for corneal opacity in anterior segment dysgenesis?**	

(Shigeto Shimmura, Yuichi Uchino, Hiroto Mitamura, Miki Omoto)

**Table:** 

**Presentation of recommendations**	

There are no reports comparing surgical treatment for corneal opacity in anterior segment dysgenesis with the natural course. Surgical treatment may provide clear corneal healing in the short term, but the long-term prognosis is unknown. Owing to the risk of complications associated with intraoperative vitrectomy and lensectomy, as well as development of postoperative secondary glaucoma, surgery is not recommended.

**Table:** 

**Strength of recommendation**	

Weak: suggest not to implement

**Table:** 

**Strength of evidence for the Clinical question**	

C (Weak)

**Table:** 

**Development process of the recommendation**	

PKP has been suggested for the surgical treatment of corneal opacity in anterior segment dysgenesis. To prepare the recommendations for this CQ, we emphasized the transparency of the transplanted cornea and postoperative visual acuity. However, the postoperative long-term prognosis is unknown, and since corneal opacity in anterior segment dysgenesis is a rare disease, no RCTs have been published to date. Therefore, the opinions of medical staff, the medical environment, and strong demands from patients and their families may have a strong influence on treatment decisions.

We conducted a literature search on the effectiveness of surgical treatment for corneal opacity in anterior segment dysgenesis. As there is no high-quality literature such as RCTs or SRs that directly compare the natural course with PKP, we included 16 case series [5, 29, 30, 32–35, 52–60], one review [18], and one case report [61]. It should be noted that there were considerable variations in age at the time of surgery, postoperative observation period, race, and disease severity in these reports. In addition, when a case series study or case report is published, there is a tendency to report relatively good clinical outcomes while poor outcomes are less likely to be published, and such publication bias needs to be considered.

The postoperative visual acuity of PKP for corneal opacity in anterior segment dysgenesis often depends on the severity of the disease. PKP is rarely performed in Japan owing to difficulties in the surgical procedure and postoperative management in infants. Additionally, in Peters anomaly, corneal opacity often improves by itself with age; however, there are no reports comparing the natural course with surgical treatment.

**Table:** 

**Summary of SR report**	

PKP has been suggested for the surgical treatment of corneal opacity in anterior segment dysgenesis. As there is no high-quality literature such as RCTs or SRs that directly compares the natural course with the postoperative course of PKP, 16 case series [5, 29, 30, 32–35, 52–60], one review [18], and one case report [61] were included. It should be noted that there were considerable variations in age at the time of surgery, postoperative observation period, race, and disease severity in these reports.

Visual acuity after keratoplasty often depends on the disease severity. In infants, the difficulty of the surgical procedure and postoperative management in keratoplasty is worth noting. Diseases with corneal opacity in anterior segment dysgenesis include Peters anomaly, sclerocornea, and anterior staphyloma. The postoperative course of PKP for Peters anomaly has been relatively well documented in a case series. In a review [18], the transparency of the cornea during the postoperative period after PKP was relatively maintained in Peters anomaly type I (corneal opacity only) but was poor in Peters anomaly type II (demonstrating ocular abnormalities other than corneal opacity). Even in case series where PKP was performed for Peters anomaly [30, 33, 54, 55], graft opacity was significantly more likely to occur in patients with ocular abnormalities other than corneal opacity and those in which rejection reaction occurred within one month after surgery [54]. Additionally, visual prognosis is significantly worse if there is glaucoma [33]. In addition to Peters anomaly, PKP for congenital corneal opacity is associated with significantly better postoperative vision in patients with bilateral opacification than unilateral opacification [32, 55]. Finally, it is reported that with long-term prognosis, postoperative 10-year corneal transparency remains at about 35% even in Peters anomaly, in which the condition of the implant is relatively stable [30].

Among conditions in which corneal opacity is caused by anterior segment dysgenesis, sclerocornea has been compared to Peters anomaly. In a case series comparing these two disease groups, the mean duration of corneal transparency was approximately 11 years in Peters anomaly and approximately three years in sclerocornea. At seven years after surgery, corneal transparency in sclerocornea is reportedly significantly worse than with Peters anomaly [29].

Studies investigating postoperative corneal transparency in case series including groups of diseases with congenital corneal opacity in addition to Peters anomaly and sclerocornea show corneal transparency of approximately 45–70% at 3–4 years after surgery [5, 35, 52]. Graft rejection occurred in approximately 40% in simultaneous cataract surgery and approximately 30% in cases without cataract surgery [52]. Graft opacity was more likely to occur in patients with repeat PKP and secondary glaucoma [35, 52]. In a multicenter study, patients who required ocular surgery other than PKP were likely to experience decreased graft transparency and postoperative visual acuity [34]. In particular, when vitrectomy and lensectomy were conducted at the same time, a significantly higher incidence of graft opacity was observed [60].

Based on the above, keratoplasty for corneal opacity in anterior segment dysgenesis often depends on disease severity, and is rarely performed in Japan owing to the difficulty of surgical procedures and postoperative management in infants. In addition, in Peters anomaly, corneal opacity itself often improves with growth, but there are no reports comparing the natural course with surgical treatment. Therefore, it cannot be determined at this time whether keratoplasty is superior to the natural course for corneal opacity in anterior segment dysgenesis. In addition, for patients whose condition is complicated with glaucoma or when lensectomy or vitrectomy is conducted at the same time as PKP, graft opacity tends to occur. Accordingly, it is necessary to carefully evaluate in advance if a patient is indicated for surgical intervention. It should be noted that the longest postoperative observation period reported to date is 10 years, and there are no detailed reports on the postoperative course after keratoplasty beyond this period.

–Ocular complications–

**Table:** 

**Clinical question 3** **What tests are useful for early detection and management of secondary ocular complications of anterior segment dysgenesis?**	

(Chie Sotozono, Yoko Ikeda)

**Table:** 

**Presentation of recommendations**	

It is necessary to understand that the criteria for detecting glaucoma in children are different from those used for adults. We suggest measurement of corneal diameter and intraocular pressure in non-crying infants and intraocular pressure and visual field tests from school age to adulthood. These are useful tests for early detection and management of secondary glaucoma in anterior segment dysgenesis. Evaluation of optic disc cupping is also important in patients whose fundus can be observed.

**Table:** 

**Strength of recommendation**	

Weak: suggest to implement

**Table:** 

**Strength of evidence for the Clinical question**	

C (Weak)

**Table:** 

**Development process of the recommendation**	

In preparing the recommendations for this CQ, we placed emphasis on avoiding overlooking secondary glaucoma. Intraocular pressure measurement needs to be conducted using multiple devices. In addition, we need to understand that the clinical findings of glaucoma in children are different from those in adults.

Since anterior segment dysgenesis is a rare disease, there are no existing clinical practice guidelines, SRs, or RCTs. There are only case series and review articles summarizing their results. However, regarding glaucoma secondary to anterior segment dysgenesis, there is a description in the pediatric glaucoma section of the Japan Glaucoma Society Guidelines for Glaucoma (4th edition), revised in 2018 [62–64].

To detect glaucoma in anterior segment dysgenesis, it is essential to measure intraocular pressure and corneal diameter in infants. For patients in whom the fundus can be observed, optic disc cupping should be evaluated. Glaucoma should be suspected when the intraocular pressure is greater than 21 mmHg in two or more measurements of intraocular pressure. However, it is often difficult to achieve appropriate measurements for various reasons. Additionally, intraocular pressure under general anesthesia tends to be measured lower than usual owing to the characteristics of children, under favorable conditions. However, the rebound tonometer, a portable tonometer that can measure without eye drop anesthesia, has a high success rate of intraocular pressure measurement without using general anesthesia or sedation in young children who are not crying [62, 65–69].

The properties of the cornea affect intraocular pressure measurements and may cause deviations from true intraocular pressure or differences between tonometers. Particularly in pediatric glaucoma, corneal diameter expansion, changes in corneal thickness, physical changes of corneal rigidity owing to corneal opacity, and corneal edema may cause errors in intraocular pressure measurements. Therefore, it is difficult to make a diagnosis based on the intraocular pressure value alone, except when markedly high intraocular pressure is observed. It is necessary to make comprehensive judgments for a pediatric glaucoma diagnosis by carefully reviewing observed findings other than intraocular pressure. Glaucoma is suspected if the corneal diameter is ≥11 mm in newborns, ≥12 mm in children aged younger than one year, ≥13 mm for all ages, or if Haab’s striae are present. It should be noted that even if there is no enlargement of the corneal diameter, eye enlargement may occur because of increased anterior chamber depth or extension of axial length. Therefore, refraction tests and axial length measurements are also considered. In adults, glaucoma is suspected when the optic cup-to-disc ratio (CD ratio) exceeds 0.7, but since the CD ratio in healthy children is small, glaucoma should be suspected when the CD ratio exceeds 0.3.

Thus, it is necessary to understand the differences in criteria for suspected glaucoma in adults and children. However, in patients with severe corneal opacity, it is often challenging to evaluate intraocular pressure or corneal diameter and fundus findings such as optic disc cupping. Findings of the anterior segment can be obtained using UBM. At the age in which children can cooperate with examinations, anterior segment OCT is useful for examinations since it is a non-contact test that takes a short time. Although UBM and anterior segment OCT are not directly useful for diagnosing glaucoma, the risk of developing glaucoma can be estimated by understanding the disease type in detail.

Intraocular pressure measurements in non-crying children, corneal diameter measurements, and evaluation of optic disc cupping are useful for early detection and management of glaucoma in anterior segment dysgenesis. When intraocular pressure measurement is difficult, it is desirable to evaluate comprehensively by measuring using multiple devices under sleeping or sedated conditions.

**Table:** 

**Summary of SR report**	

1. **Tests useful for early detection of secondary ocular complications**

A literature search was conducted for clinical practice guidelines, reviews/commentaries, and case reports for anterior segment dysgenesis, including Peters anomaly, sclerocornea, aniridia, Axenfeld anomaly, corneal opacity, pediatric glaucoma, and pediatric secondary glaucoma. The most important tests are intraocular pressure, corneal diameter, and observation of fundus findings (optic disc cupping). It is recommended to measure intraocular pressure using multiple devices, and it is desirable to perform a comprehensive evaluation using different methods such as the rebound tonometer, Perkins applanation tonometer, Tono-Pen^®^, and palpation [70]. Intraocular pressure measured under general anesthesia results in readings that are generally lower than the actual values [71, 72]. Glaucoma should be suspected when the intraocular pressure is >21 mmHg for two or more measurements [73, 74]. In addition, especially until they are approximately two years old, increased corneal diameter accompanied by eye enlargement can be seen when intraocular pressure increases. Therefore, measurement of corneal diameter may be important for early detection. Glaucoma can be suspected if the corneal diameter is ≥11 mm in newborns, ≥12 mm in infants aged younger than 1 year, ≥13 mm for all ages, or whenever Haab’s striae are present. Even if enlargement of the corneal diameter is not noticeable, anterior chamber depth and axial length may need to be measured as they can cause eye enlargement. Optic disc cupping typically expands in a concentric circle. If the CD ratio is >0.3 or there is progression of indented areas, or if there is a difference between the left and right eyes, glaucoma should be suspected [75]. Similarly, for adult patients with anterior segment dysgenesis, intraocular pressure should be measured with multiple devices and follow-up should be performed. If the fundus can be observed, it is also useful for early glaucoma detection by regularly monitoring the thinning of the retinal nerve fiber layer using posterior segment OCT. Besides visual fundus mapping, the RetCam handheld wide-angle imaging apparatus is useful in infants [76], whereas a fundus camera is useful in school aged children and adults to record fundus findings.

If corneal opacity is present and visualization of the anterior segment is poor, then UBM is useful [22]. This instrument can help evaluate the anterior chamber angle (anterior chamber depth, open-angle or presence of obstructed angle, its extent), corneal thickness, posterior corneal defect, strands arising from the iris, malposition of the lens, and the iris. In addition to UBM, anterior segment OCT can also be used in adults and in children at the age where they can cooperate with examinations. Anterior segment OCT is useful as it requires less contact than UBM and has a short examination time.

2. **Tests useful for detection of secondary ocular complications**

The visual field cannot be measured in infants, and OCT, too, cannot be used in many cases. Therefore, comparisons with initial findings are essential for managing glaucoma. Findings of intraocular pressure, refraction, corneal diameter, corneal thickness, axial length, CD ratio, retina, and anterior chamber depth and angle by UBM should be obtained and recorded if possible. It is desirable to take photographs of the anterior segment, angle, fundus, etc., and maintain records where possible.

In addition to understanding the test items necessary for early detection and management, it is important to understand that intraocular pressure, axial length, refraction, and optic disc cupping during childhood development have normal values and characteristics different from those for adults. Since intraocular pressure increases when children are crying, it is necessary to measure it when they are not crying. The rebound tonometer has a high success rate of intraocular pressure measurement without using general anesthesia or sedation in young non-crying children. However, if intraocular pressure measurement is difficult or when anterior segment and fundus examinations cannot be performed, examinations under sleeping or sedated conditions are required. If oral or suppository sedatives do not provide sufficient sedation, general anesthesia should be considered. If examinations under general anesthesia are possible, all necessary tests such as refraction, corneal diameter, corneal thickness, axial length, anterior chamber/angle findings, and fundus examination should be performed in addition to intraocular pressure.

From school age to adulthood, periodic intraocular pressure measurements are useful for early glaucoma detection. In anterior segment dysgenesis, it is difficult to measure intraocular pressure accurately owing to the irregular corneal surface; hence, it is desirable to measure intraocular pressure using multiple devices.

3. **Tests useful for management of secondary ocular complications**

As the focus is on glaucoma as a secondary ocular complication of anterior segment dysgenesis, the most important test for management is intraocular pressure. When glaucoma is diagnosed based on high intraocular pressure, treatment (eye drops, surgery) to control intraocular pressure is performed. Progress is monitored to ensure that intraocular pressure is well controlled. Among the case reports, there was a report showing that the axial length of only one eye was increased despite good control of intraocular pressure within the normal range after glaucoma surgery [77]. Similar to cases where corneal diameter may increase when intraocular pressure rises in children up to approximately two years of age [22, 78], a compensatory increase of axial length may have occurred, resulting in false normal values. For this reason, periodic corneal diameter and axial length measurements are considered useful for management [78, 79]. Myopia associated with extension of axial length affects uncorrected visual acuity. Some patients require refraction correction and prosthetics to support changes in uncorrected visual acuity and refraction. In patients where the cornea becomes cloudy and the angle cannot be observed, it is useful to measure using UBM and compare data with the previous angle condition if intraocular pressure increases [22]. Additionally, even if intraocular pressure is maintained within the normal range, progress of glaucoma should be suspected if expansion of optic disc cupping is seen. Therefore, periodic evaluations of optic disc cupping and visual field tests are necessary.

4. **Management of patients without glaucoma at present**

Once the anterior segment dysgenesis disease type is diagnosed, the approximate probability of developing glaucoma may be estimated [80–84]. Even if patients currently do not have glaucoma, this does not imply that they will not develop it in the future; it is necessary to keep the possibility of glaucoma in mind and conduct periodic examinations even into adulthood.

Axial length, corneal diameter, and intraocular pressure change over time with growth. Therefore, it is necessary to understand normal values for each age range [71, 78, 79]. In addition, it is necessary to understand that the evaluation of optic disc cupping is different from adults[85]. Since intraocular pressure is higher when children are crying, it is necessary to measure intraocular pressure at rest under sleeping or sedated conditions if measurement with rebound tonometers, etc., is difficult. If oral administration of sedatives or suppositories does not induce sufficient sleep, general anesthesia should be considered. If examinations under general anesthesia are conducted, all necessary tests such as refraction, corneal diameter, corneal thickness, axial length, anterior chamber/angle findings, and fundus examination should be performed in addition to intraocular pressure.

From school age onwards and in adults, periodic intraocular pressure measurements, visual field tests, and, if possible, evaluation of optic disc cupping are required for glaucoma management. If the corneal surface is irregular because of anterior segment dysgenesis, it will be difficult to measure intraocular pressure accurately; therefore, measurements should be taken using multiple devices.

Adverse events due to the use of intraocular pressure measurement devices, UBM, and OCT in the diagnosis of anterior segment dysgenesis are not reported in any literature reviewed at this time. The risk from use of local anesthesia for intraocular pressure measurement and UBM is low, and the risk from use of general anesthesia is low if performed under appropriate management.

## Abbreviations

CD: Cell density
CQ: Clinical question
MA: Meta-analysis
OCT: Optical coherence tomography
PKP: Penetrating keratoplasty
RCT: Randomized controlled trial
SR: Systematic review
UBM: Ultrasound biomicroscopy
