# Correction: Clinical practice guideline for anterior segment dysgenesis

**DOI:** 10.1007/s10384-026-01385-6

**Published:** 2026-07-03

**Authors:** Research on rare and intractable diseases, Health, Labour and Welfare Sciences Research Grants Clinical Practice Guideline Development Committee for Anterior segment dysgenesis of the “Research group on establishing standardized diagnosis and treatment of Intractable corneal diseases”

**Affiliations:** Osaka, Japan

**Correction: Jpn J Ophthalmol. 2026;70(2):472–492** 10.1007/s10384-025-01297-x

In this article, the author group was incorrectly given as: ‘“Research on rare and intractable diseases, Health, Labour and Welfare Sciences Research Grants Clinical Practice Guideline Development Committee for Aniridia of the “Research group on establishing standardized diagnosis and treatment of Intractable corneal diseases”’ but should have been: ‘Research on rare and intractable diseases, Health, Labour and Welfare Sciences Research Grants Clinical Practice Guideline Development Committee for Anterior segment dysgenesis of the “Research group on establishing standardized diagnosis and treatment of Intractable corneal diseases”’.

